# Dog Breeding in Italy: A Technical Overview of Husbandry and Reproductive Management

**DOI:** 10.1002/vms3.71198

**Published:** 2026-08-23

**Authors:** Lluis Ferré‐Dolcet, Francesco Petronella, Giovanni Bramante, Alice Carbonari, Lorenza Frattina, Olga Maria Andriulo, Vincenzo Cicirelli

**Affiliations:** ^1^ AniCura ARS Veterinaria SLP Barcelona Spain; ^2^ Department of Veterinary Medicine University of Bari Aldo Moro Valenzano Bari Italy

**Keywords:** canine reproduction, Italian breeders, kennels, oestrus monitoring, progesterone, puppy management

## Abstract

**Background:**

Appropriate reproductive management is essential for improving pregnancy outcomes and neonatal survival in dogs. However, information on the practices adopted by Italian dog breeders remains limited.

**Objectives:**

This study aimed to describe reproductive and neonatal management practices among certified Italian dog breeders, compare them across Northern, Central and Southern Italy, and assess their consistency with current veterinary recommendations.

**Methods:**

An anonymous online questionnaire comprising 41 questions was distributed to Italian dog breeders between October 2023 and March 2024. It collected information on oestrus monitoring, mating and artificial insemination, semen type, pregnancy diagnosis and puppy management until weaning. Of all questionnaires received, 251 met the inclusion criteria.

**Results:**

The study included 154 breeders from Northern, 49 from Central and 48 from Southern Italy. Management practices were generally homogeneous across areas. Progesterone measurement was used by 96.4% of breeders, although approximately 70% were unaware of the analytical method employed. Natural mating and fresh semen were the preferred reproductive options. Ultrasonography was widely used for pregnancy diagnosis, performed at 26.55 ± 4.84 days after expected ovulation, whereas 87% of breeders requested radiographic foetal counting. Regional differences concerned oestrus detection, diagnostic testing, mating intervals, radiographic timing and puppy weighing frequency. Northern breeders weighed puppies more frequently than Central and Southern breeders (*p* < 0.001).

**Conclusion:**

Italian breeders generally follow established reproductive and neonatal practices. Nevertheless, targeted education on progesterone assay interpretation, vaginal cytology and advanced reproductive technologies could improve and standardize breeding management.

## Graphical abstract

Italian dog breeders completed a nationwide questionnaire on reproductive management. Macro area comparisons of oestrus detection, mating and neonatal care revealed consistent practices but highlighted gaps in breeder education.

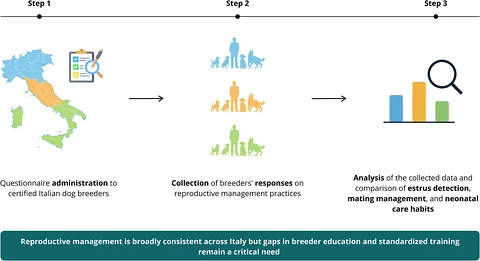

## 1 Introduction

Canine breeding is a globally expanding business, despite the socio‐sanitary challenges posed by canine overpopulation and the increasing number of stray dogs in many countries (Dotson and Hyatt 2008). Following the COVID‐19 pandemic, the market for companion dogs grew significantly (Siettou 2021). Although there are no official data on the number of breeders in Italy in 2024, an estimate based on ENCI (Ente Nazionale Cinofilia Italiana) data suggests that there are approximately 1700 registered kennels. Successful pregnancy in bitches depends largely on appropriate breeding soundness examinations and accurate evaluation of the oestrus cycle. Ovulation timing is a standard procedure used to identify the optimal moment for natural mating or artificial insemination, thereby increasing pregnancy success rates and predicting parturition timing (Barstow et al. 2018). In small animal practice, commonly used methods include behavioural observation, serum progesterone (P4) assays, vaginal cytology and ovarian ultrasonography (Fáy et al. 2003). Although some of these techniques lack reliability when used alone, combining them can improve the accuracy of ovulation prediction. Additional methods, such as vaginoscopy, LH peak measurement and vaginal electrical impedance, have also been studied but are considered either unreliable or, in the case of LH measurement, difficult to apply in practice (Bergeron et al. 2014; Guérin et al. 1997; Jeffcoate and Lindsay 1989). For this reason, progesterone level determination is often complemented with vaginal cytology and ovarian ultrasonography to improve diagnostic accuracy. During pregnancy, various diagnostic tools can help evaluate foetal viability, detect abnormalities and estimate the number of foetuses. These techniques are essential in minimizing complications during gestation and parturition. Several recent surveys have examined breeding practices among dog breeders during reproductive, gestational and post‐partum phases (Blackman et al. 2020; Santos dos et al. 2020; Santos et al. 2021). This study examines the trends of Italian breeders to highlight any critical points. To date, on the basis of internet and training programme research, educational programmes for canine reproduction directed to breeders are lacking in Italy. Breeders’ experience can sometimes reduce the perceived need for veterinary support; however, poor reproductive management can result in technical issues, economic losses if pregnancy is not achieved and increased veterinary costs in case of complications.

### 1.1 Aim

This study aims to describe the reproductive management practices and breeding techniques used by Italian dog breeders. These data will compare breeders’ performances across the North, Centre, and South of the country, identifying management challenges and evaluating them in relation to current recommendations from the recent scientific literature. By offering a descriptive overview of current practices, the study seeks to highlight the main critical points in Italian dog breeding and provides a basis for informed, science‐driven improvements.

## 2 Materials and Methods

The present study was conducted in the whole Italian territory. Certified canine breeders by ENCI (Italian National Kennel Club) were recruited at dog shows and through social media breeder's groups and invited by email to complete an anonymous online questionnaire on breeding management practices. The inclusion criteria for this study included dog breeders currently operating in the Italian territory who voluntarily agreed to participate, acknowledged the confidentiality and anonymity of their responses and fully completed the online questionnaire about their breeding management practices. Data collection was performed from October 2023 to March 2024. The questionnaire was administered with the aim of covering the entire Italian territory. The questionnaire consisted of 41 questions requiring short or numerical answers about where the breeding facility was located, how many breeds were kept in the facility for breeding purposes, and how the heats, pregnancies and newborn puppies were managed (how heats were monitored and with which techniques, if artificial insemination was performed, if pregnancy diagnosis and follow‐up was done, and how newborn puppies were managed until weaning). Once the questionnaires were completed, data were analysed, and incomplete questionnaires were removed from the study. As questionnaires were anonymous, breeders could not be contacted in the case of incomplete or incomprehensible information provided. Incomplete questionnaires, those with contradictory or unintelligible information, were excluded from the final analysis.

Using the sample size calculator (Qualtrics), the number of respondents was established to ensure that the sample is adequate and representative of Italian dog breeders. As no animals were manipulated for data collection, no ethical committee approval was needed.

### 2.1 Statistical Analysis

All data collected through the questionnaire were entered into Microsoft Excel and subsequently analysed using RStudio software (version 2025.09.0). Descriptive statistics were calculated for all categorical and continuous variables. The categorical variables were summarized as absolute and relative frequencies (percentages), whereas continuous variables were reported as means ± standard deviations (SD). The distribution of each continuous variable was assessed for normality using Shapiro–Wilk test, and the homogeneity of variances was evaluated using either Bartlett's test (for normally distributed data) or Levene's test (for non‐normally distributed data). To investigate regional differences, breeders were grouped into three geographical macro areas: North (*Valle d'Aosta, Piemonte, Liguria, Lombardia, Trentino‐Alto Adige, Veneto, Friuli‐Venezia Giulia* and *Emilia‐Romagna*), Centre (*Toscana, Umbria, Marche, Lazio, Abruzzo* and *Molise*) and South (*Campania, Puglia, Basilicata, Calabria, Sicilia* and *Sardegna*). For categorical variables, contingency tables were constructed, and the chi‐square test of independence (*χ* ^2^) was applied to assess associations between each variable and the geographical macro area. When the expected frequency in any cell was below 5, Fisher's exact test was considered for validation. Continuous variables were compared among macro areas using the Kruskal–Wallis test. When significant differences were detected, appropriate post hoc analyses were conducted using the Dunn test for pairwise comparisons with Bonferroni or Holm correction when appropriate. A *p* value lower than 0.05 was considered statistically significant.

## 3 Results

### 3.1 Breeder and Breed Distribution

**FIGURE 1:**
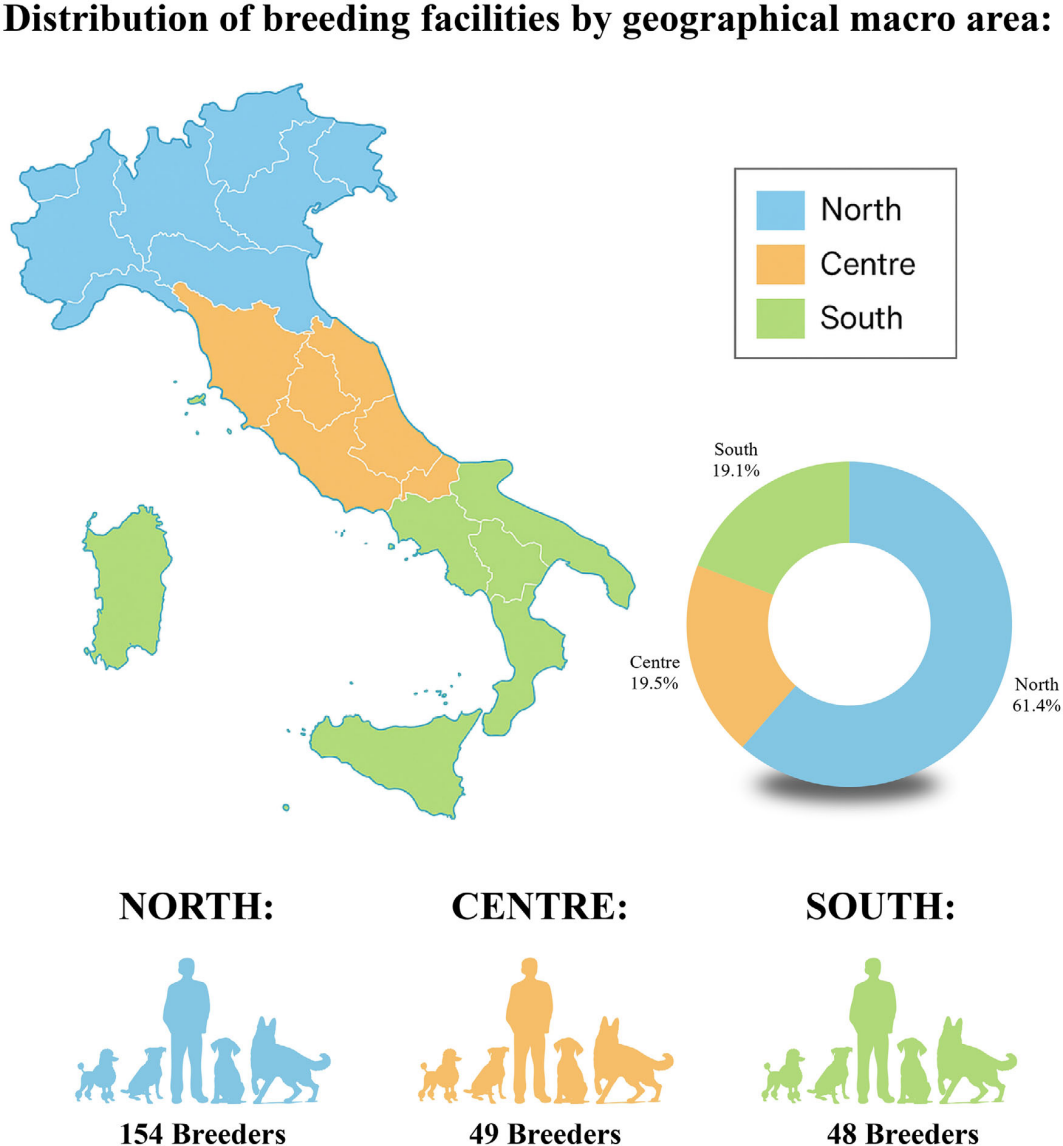
Geographical distribution of the breeders included in the study (*n* = 251) across Italy, classified according to the macro areas described in the text: 154 breeders in Northern Italy (blue), 49 in Central Italy (yellow), and 48 in Southern Italy (green). The pie chart illustrates, using the same colour scheme, the proportional representation of breeders within each macro area.

**FIGURE 2:**
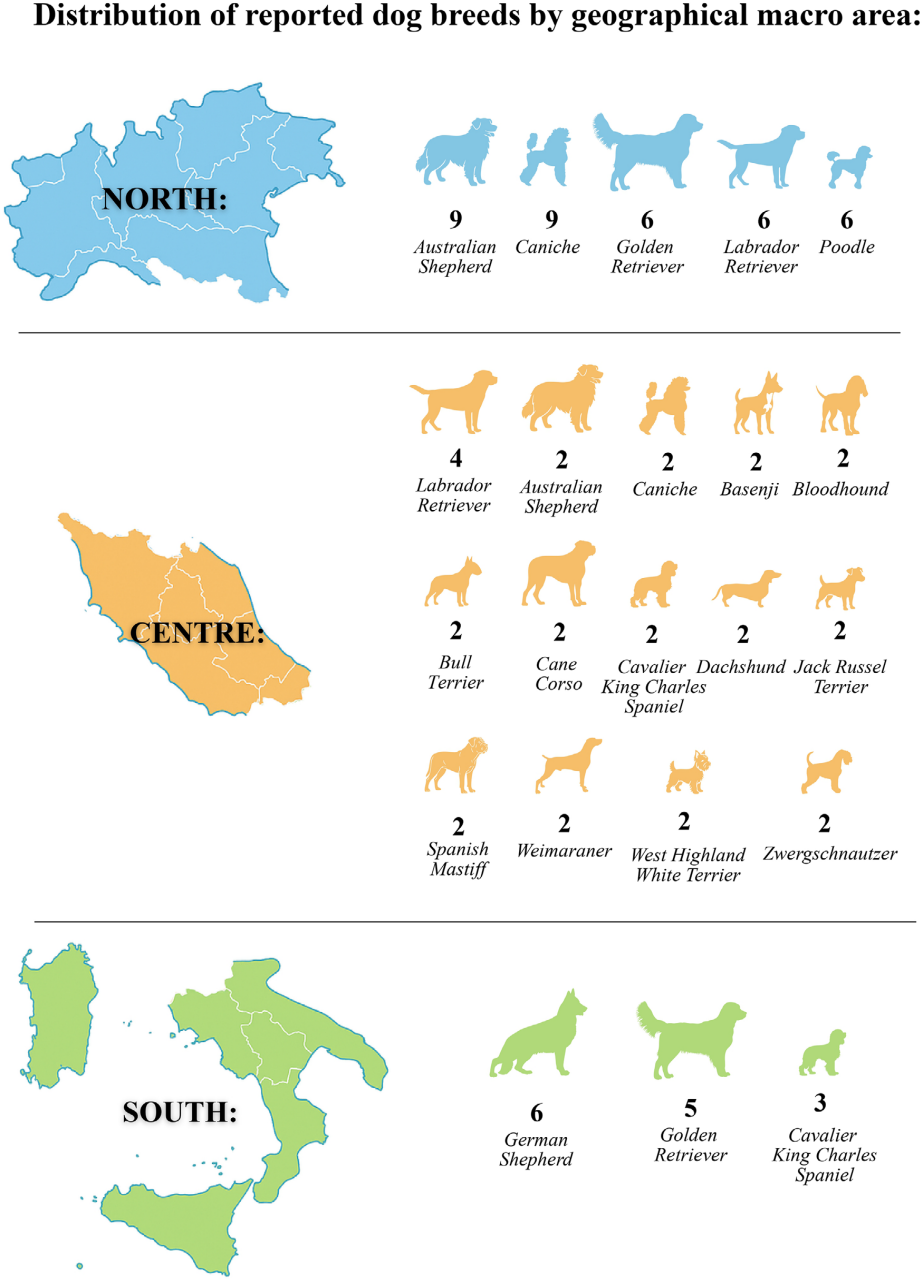
Distribution of the main breeds of the kennels considered in the study.

On the basis of the study's inclusion criteria, questionnaires from a total of 251 Italian breeders were analysed. One limitation of the study was the inability to obtain an equal number of respondents across the three macro areas (North, Centre and South). This was mainly due to the fact that most questionnaires were distributed to breeders attending dog shows in Northern Italy, whereas breeders in Central and Southern Italy were contacted exclusively via email. Participants were distributed across the three main geographical macro areas: 154 breeders (61.4%) from Northern Italy, 49 (19.5%) from Central Italy and 48 (19.1%) from Southern Italy, as shown in Figure 1. Overall, the most frequently reported breeds were the Australian Shepherd (*n* = 13), Labrador Retriever (*n* = 12), Golden Retriever (*n* = 11), Poodle (*n* = 11) and Cavalier King Charles Spaniel (*n* = 10). Figure 2 shows the distribution of the main breeds raised in the different macro areas.

### 3.2 Reproductive Management Practices

**FIGURE 3:**
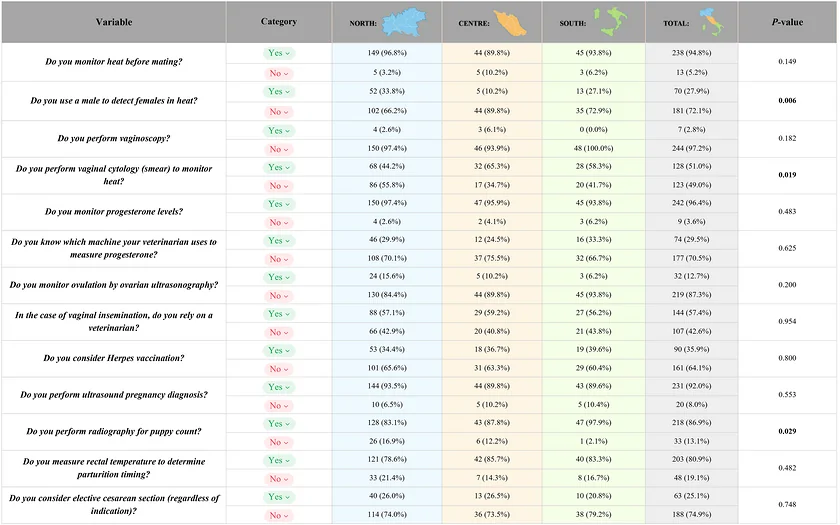
Tabular representation of the frequency distribution (%) of categorical reproductive management variables across the geographical macro areas. *p* values presented in bold indicate statistically significant differences.

**FIGURE 4:**
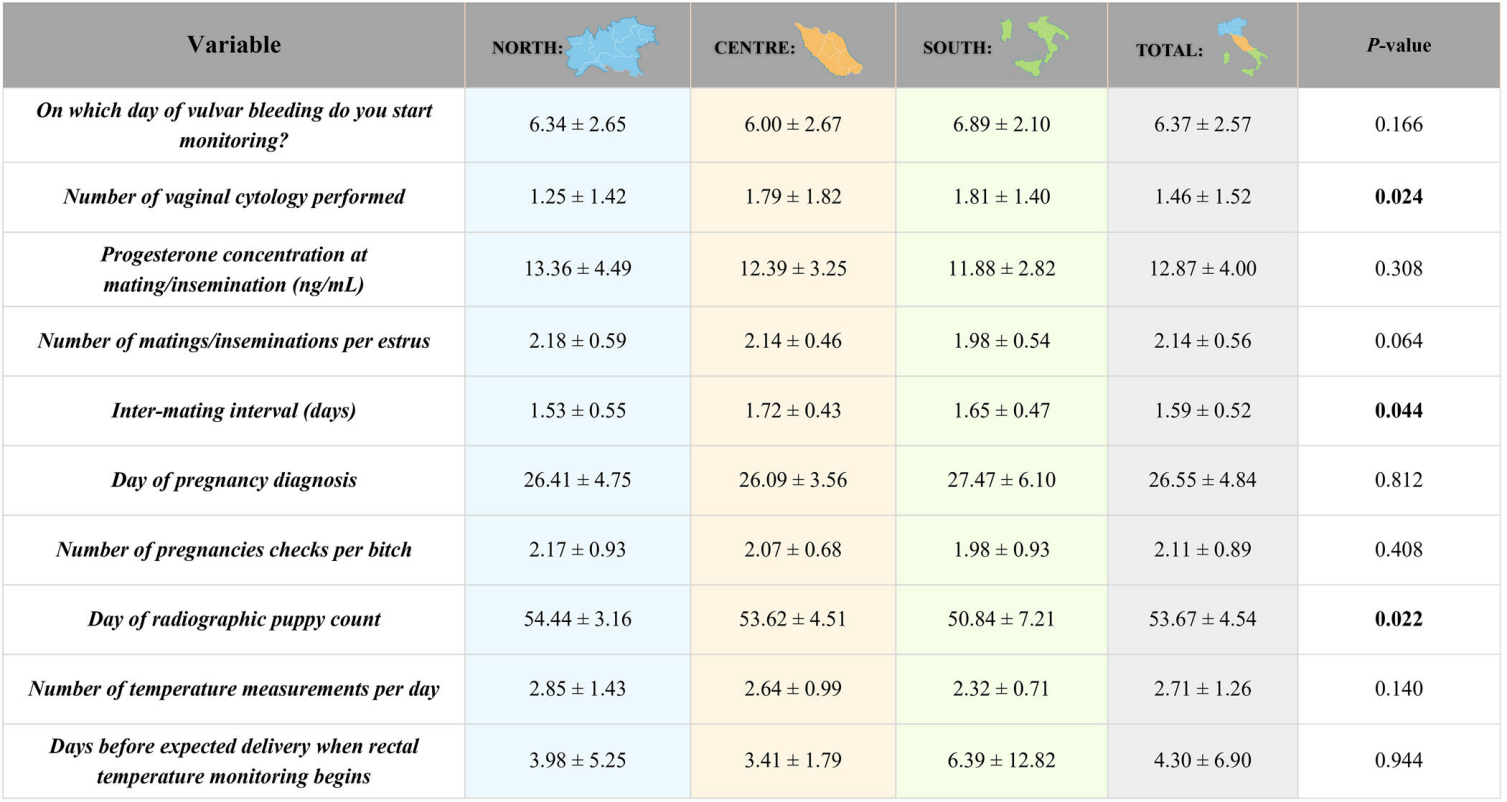
Tabular representation of descriptive statistics (mean ± standard deviation) of continuous reproductive management variables across the geographical macro areas. *p* values presented in bold indicate statistically significant differences.

**FIGURE 5:**
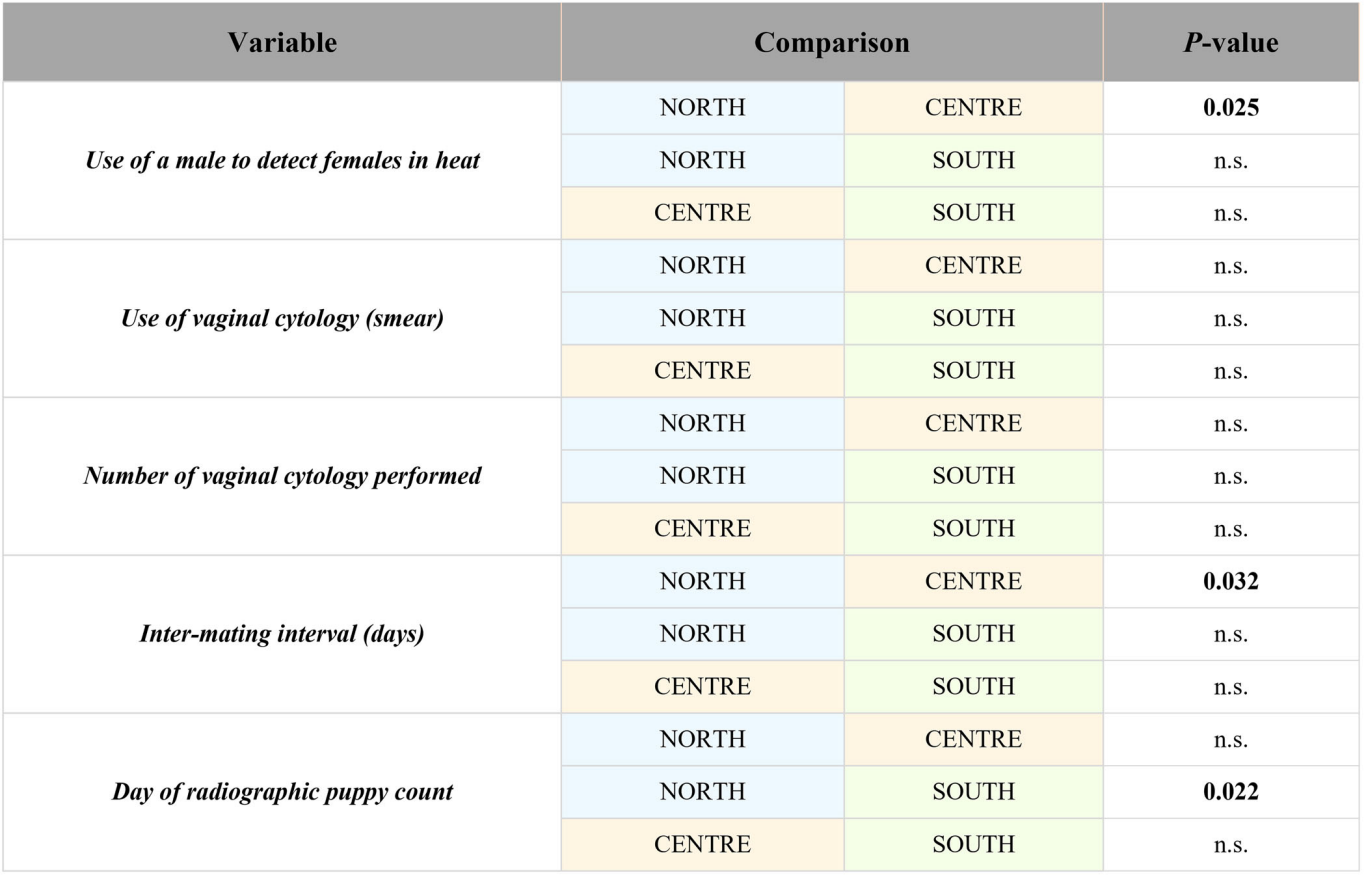
Post hoc pairwise comparisons for variables showing significant differences. Comparisons were conducted between North and Centre, North and South, and Centre and South. *p* values presented in bold indicate statistically significant differences, whereas ‘ns’ denotes the absence of a statistically significant difference.

The results concerning reproductive management are presented in Figures 3, 4, 5. Overall, breeders showed generally homogeneous reproductive management practices across the three geographical macro areas. Most respondents routinely monitored oestrus and P4 concentrations and reported similar mating methods and pregnancy‐monitoring practices across the three macro areas. Significant differences were observed for two categorical variables: the use of a male to detect females in heat and the use of vaginal cytology to monitor oestrus. Both showed regional variation in the overall distribution. However, post hoc analyses revealed that only the use of a male was statistically significant after Bonferroni correction, whereas vaginal cytology did not show any significant pairwise differences, indicating that the discrepancies were modest and not regionally structured.

Among continuous variables, differences were detected in the approximate number of tests performed, the inter‐mating interval and the day of radiographic puppy count. Post hoc comparisons showed that breeders from Central and Southern Italy reported performing a higher number of diagnostic tests than those from Northern regions. The inter‐mating interval tended to be slightly longer in Central Italy, whereas radiographic puppy counting was carried out earlier in Southern Italy (50.84 days) than in the other regions (54.94 North and 53.62 Centre) (Figure 4).

**FIGURE 6:**
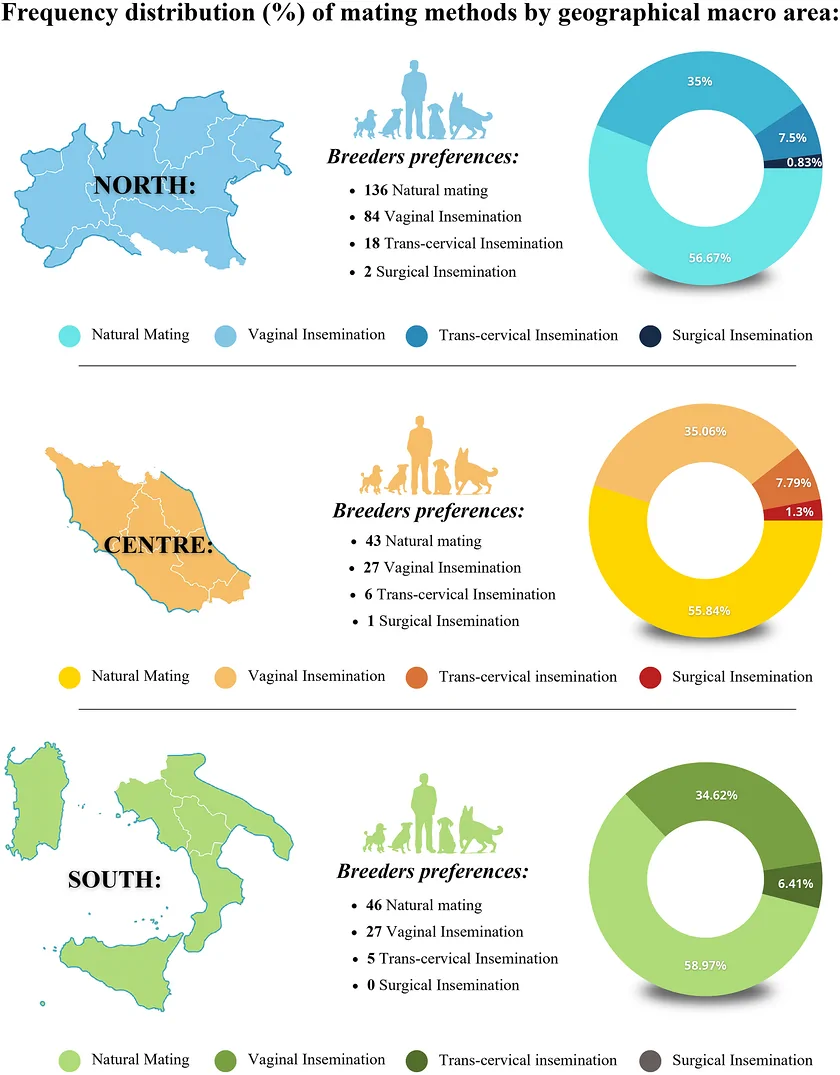
Distribution of breeders’ mating preferences across geographical macro areas (North, Centre and South). Each breeder could select more than one option. The total number of preferences reported for each mating method is indicated, and the corresponding percentage is illustrated using pie charts for each macro area.

Regarding the most used mating technique in the various macro areas, breeders reported various methods in order of usefulness, including natural mating, vaginal insemination (VI), trans‐cervical insemination (TCI) and surgical insemination. Figure 6 presents the different techniques employed by breeders across the macro areas, along with their corresponding frequencies. No statistically significant differences were observed between the macro areas, suggesting that the choice of mating method is not influenced by geographical location. The percentages of use of the different techniques in the three macro areas are comparable. However, the surgical insemination technique has not been reported to be used in the south.

**FIGURE 7:**
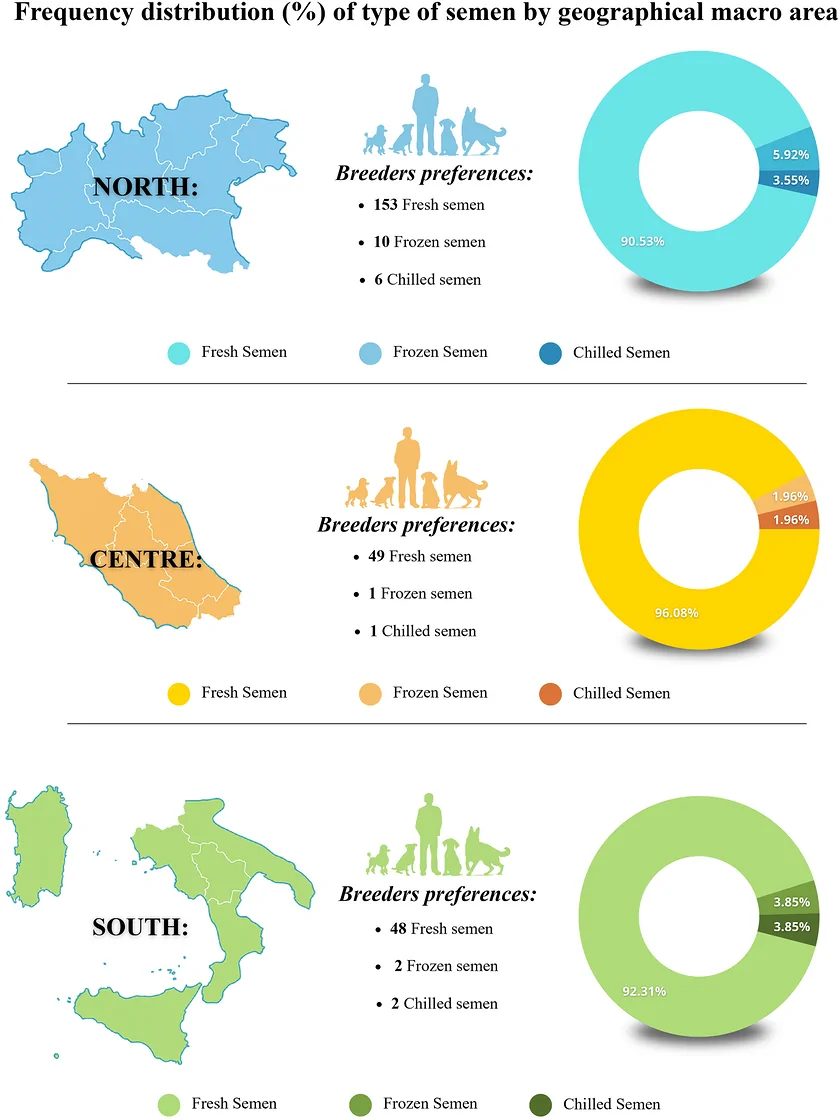
Distribution of breeders’ semen preferences across geographical macro areas (North, Centre and South). Each breeder could select more than one option. The total number of preferences reported for each type of semen is indicated, and the corresponding percentage is illustrated using pie charts for each macro area.

About the most used semen type in the various macro areas, breeders reported the usage of fresh, frozen and chilled semen. Figure 7 presents breeders’ semen type preferences along with the corresponding frequencies. There were no statistically significant differences observed in the semen used between macro areas. Overall, for all macro areas, the most frequently used type of semen was fresh semen (*p* < 0.05).

### 3.3 Puppy Management Practices

**FIGURE 8:**
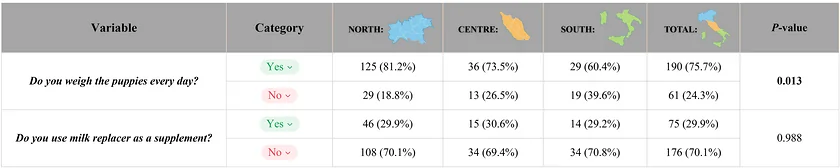
Tabular representation of the frequency distribution (%) of categorical puppy management variables across the geographical macro areas. *p* values presented in bold indicate statistically significant differences.

**FIGURE 9:**
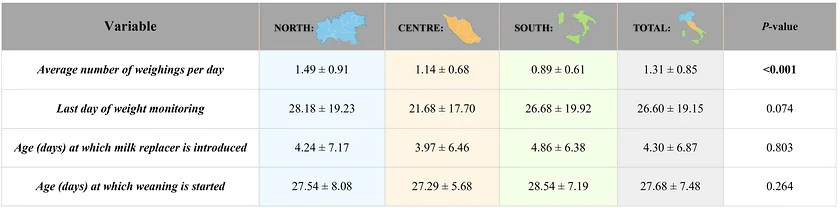
Tabular representation of descriptive statistics (mean ± standard deviation) of continuous puppy management variables across the geographical macro areas. *p* values presented in bold indicate statistically significant differences.

The results concerning puppy management are summarized in Figures 8 and 9. Overall, breeders reported largely consistent practices across the three geographical macro areas. Most respondents stated that they routinely weighed puppies to monitor growth and followed similar protocols for feeding and weaning. The use of milk replacer as a dietary supplement was relatively uncommon, with limited regional variation observed.

A statistically significant difference was identified for the average number of daily weightings (*p* < 0.001). Breeders from Northern Italy reported weighing puppies more frequently compared to those from Central and Southern regions. Post hoc comparisons confirmed significant differences between all area pairs (North–Centre, North–South and Centre–South) (*p* < 0.001). For the categorical variable ‘Do you weigh the puppies every day?’, a difference among macro areas was also detected (*p* = 0.023). Moreover, post hoc pairwise comparisons using the Holm correction revealed a statistically significant difference between breeders from North and South macro areas (*p* = 0.018), whereas no significant differences were observed between the other area pairs (North–Centre and Centre–South). No other variables showed statistically significant differences among the geographical areas. These findings indicate a generally homogeneous approach to early‐life puppy management throughout the country, with regional variability limited primarily to the intensity of daily growth monitoring.

## 4 Discussion

To the author's knowledge, this is the first descriptive study that documents canine breeders’ approach to reproductive performance in a Southern European country.

This nationwide survey of 251 Italian dog breeders offers a comprehensive snapshot of current reproductive and early‐life management practices across Northern, Central and Southern Italy. Overall, breeders reported mostly consistent approaches to oestrus monitoring, pregnancy diagnosis and puppy care, with regional differences mainly in how intensely and when they conduct monitoring rather than in their core practices.

### 4.1 Breeder and Breed Distribution

Although there are no official data on the number of breeders in Italy in 2024, an estimate based on ENCI data suggests that there are approximately 1700 registered kennels. Dividing Italy into three macro areas, showing the number of kennels and the most representative breeds in the study, made it easier to interpret the data, providing a satisfactory overall view (Figures 1 and 2).

### 4.2 Heat Detection Techniques

For both veterinarians and breeders, the identification of the optimal day of mating is crucial to achieve a higher pregnancy rate. This study highlights that all three macro areas show a substantially overlapping trend in preferences in the use of the different techniques employed to identify the optimal moment for mating of the female. The canine oestrous period is longer than in other species, and clinical symptoms during the oestrus cycle stages are non‐specific. The whole heat (pro‐oestrus and oestrus) can last 3–4 weeks, whereas the best time for mating is only 3–4 days (Concannon and Rendano 1983). The female can accept the male even in pro‐oestrus, thus causing the fertilization of the gametes to fail. In this study, data show that the use of the male to monitor oestrus is falling out of use throughout Italy. In fact, all the breeders answered use progesterone to monitor the bitch. Determining the LH peak requires several blood samples each day until its detection, whereas P4 increases progressively during heat (Hase et al. 2000). Together with the longer oestrus cycle, another issue in canine reproduction is that oocytes are immature just after ovulation and need around 48–72 h for complete maturation and for being fertilizable (Chastant‐Maillard et al. 2011; Reynaud et al. 2005). Ovulation detection is crucial for good reproductive management. Therefore, P4 currently seems to be the most accurate and easily detectable hormone to estimate ovulation. However, there is still no single P4 concentration value that definitively determines ovulation in bitches, as P4 concentrations vary significantly depending on the measurement method used (Gloria et al. 2018; Nöthling and De Cramer 2018; Zuercher et al. 2021). The near‐universal P4 monitoring in our cohort study (96.4%) underscores the role of hormonal assays in breeding management even if around 70% of breeders do not know the detection method used. Although this may not be critical since it is the veterinarian's responsibility to understand the analytical system to correctly identify ovulation, it is interesting to note that the average P4 concentration when the breeder decided to mate the bitch or perform artificial insemination was around 12.8 ± 4.00 ng/mL. Even if most of breeders did not know what machine were their vets using for P4 measurement, these results are in accordance of the usage of a chemiluminescence or fluorescence assay, as ovulation tends to be detected between 4 and 10 ng/mL (Brugger et al. 2011; Gloria et al. 2018; Milani et al. 2022; Rota et al. 2016; Tal et al. 2020). The high prevalence detected on P4 assays reflects the actual clinical recommendations that emphasize P4 as the key marker for ovulation detection for insemination optimization. On the other side, comparative evaluations of P4 machines (RIA vs. chemiluminescence/FEIA/CLIA) showed that modern CLIA/chemiluminescent assays correlate well with good‐standard methods, support high reliability when used appropriately (Milani et al. 2022) but may require assay‐specific thresholds for clinical decision‐making (Nöthling and De Cramer 2018, 2019; Tal et al. 2020). This variability underscores why progesterone values alone should not be interpreted without knowledge of the specific assay used, a point of practical relevance for both clinicians and breeders. On the basis of questionnaire responses, vaginal cytology was performed and evaluated by the veterinarian in approximately by half of respondents with modest regional variations starting between the second and third day of vulvar discharge with an average of two vaginal smears (Figures 3 and 4). More than 50% of breeders using vaginal cytology as a test for oestrus staging consider this technique valuable during oestrus monitoring (Figure 4). Vaginal cytology remains an inexpensive and fast in‐clinic technique for oestrus monitoring, often used together with P4 measurements. Despite breeders’ generally positive perception of this technique, vaginal cytology interpretation is performed by the veterinarian and requires specific training; the literature reports poor inter‐observer agreement on vaginal cytology cell‐type classification (Moxon et al. 2010; Reckers et al. 2022) suggesting that breeders’ favourable view of this tool may not fully reflect its technical reliability, and that it should always be complemented by hormonal data. In fact, the proportion of breeders using vaginal cytology in our study is consistent with reports that many practitioners and breeders combine cytology and P4 for optimal timing (Barstow et al. 2018; Fáy et al. 2003; Roos‐Pichenot and Zakošek Pipan 2025). Vaginoscopy was the least used technique, as almost 90% of breeders considered it useless. As physiological background, both techniques, vaginal cytology and vaginoscopy, might be useful to estimate the oestrus cycle phase: The rise in oestradiol during pro‐oestrus increases vaginal mucosa swelling and crenulation, and a progressive increase in keratinized cells occurs as oestrus approaches. Once the oestrus phase is reached, more than 90% of epithelial cells are superficial keratinized cells, most with pyknotic nuclei (Bouchard et al. 1991). Nevertheless, both vaginoscopy and vaginal cytology, whether used alone or combined, are unable to determine the exact moment of ovulation. The comparison of mating practices between regions (Figure 5) does not show marked differences among the macro areas considered. It must be considered that up to 80% of breeding failures in the canine population are attributed to incorrect timing of mating or insemination (Roos‐Pichenot and Zakošek Pipan 2025). Breeders’ educational programmes are often lacking across Europe, and no harmonized regulatory approach to breeder training as yet been established.

### 4.3 Mating and Type of Semen Preferences

Over all the responses received, natural mating remained the predominant method across all the macro areas studied (>55%). VI was used by only a third of the respondents, whereas TCI was uncommon and surgical insemination was absent in the South of Italy and rare in central and northern Italy (Figure 6). These distributions mirror wider clinical practice where natural mating and VI with fresh semen remain common, whereas TCI and surgical intrauterine techniques are commonly used primarily for chilled or frozen semen (Romagnoli and Lopate 2014; Mason 2018).

The strong preference for fresh semen across regions (>90%) is consistent with established evidence showing higher conception rates with fresh semen compared with frozen or chilled semen when insemination is optimally timed (Figure 7). The low use of frozen semen reported by breeders in our sample may reflect their concern about the additional costs and logistical demands associated with TCI, which is generally required to achieve adequate conception rates with frozen‐thawed semen (Cicirelli et al. 2021; Hollinshead and Hanlon 2017; Linde‐Forsberg et al. 1999; Silva et al. 1995; Thomassen et al. 2006). Although well‐timed TCI performed by trained clinicians can yield satisfactory pregnancy rates even with frozen semen, the higher cost and need for specialized expertise likely discourage breeders from adopting this approach on a routine basis. These technical and logistical demands might likely explain the sustained preference for semen among breeders (Joonè 2024; Linde‐Forsberg et al. 1999). Nevertheless, several reports indicate higher pregnancy rates with well‐timed TCI using frozen semen in specific settings and breeds, highlighting that TCI provides an effective, non‐surgical intrauterine option when performed by trained clinicians (Pretzer et al. 2006). Nonetheless, logistical cost and training barriers limit TCI adoption at the breeder level. The limited demand for frozen and chilled semen suggests that the use of imported genetic material remains relatively low in Italy. This fact is of particular interest as, over time, this restricted genetic exchange may contribute to reduced genetic diversity within breeds, increasing the risk of inbreeding and limiting the introduction of desirable reproductive or health‐related traits.

### 4.4 Puppy Management Practices

Pregnancy diagnosis was performed on average at the age of 26.55 ± 4.84 days from expected ovulation (Figure 4) with ultrasonography widely adopted (>90%), consistent with its recognized role in early, non‐invasive pregnancy confirmation (Lopate 2018). Most of the breeders (87%) demanded the x‐ray puppy count, with Southern breeders performing x‐rays slightly earlier on average, reflecting different scheduling practices or a tendency for early imaging to plan whelping logistics, but both ranges fall within the commonly accepted window for reliable foetal skeletal visualization (Concannon and Rendano 1983). The timing differences, while statistically significant, were modest, and their clinical relevance should be interpreted with caution. In fact, earlier radiographs may yield less accurate foetal counts if the gestational age is inadequate. Interestingly, the questionnaire responses showed that breeders in Central and Southern Italy reported performing fewer diagnostic tests overall than breeders in Northern Italy. This difference may reflect regional variations in access to specialized reproductive services (Santos et al. 2021).

Routine weighing of puppies to monitor growth was common, but frequency varied regionally: Northern breeders weighed puppies more often compared to Central and Southern (Figures 8 and 9). Daily increase in weight is significant for puppy survival, especially in low‐weight puppies at birth, and to detect early failure to thrive, guide supplementary feeding and improve survival (Mugnier et al. 2023). Thus, regular weighing is clue to monitor puppies’ growth and detect early problems. Daily weighing should be performed during the first 2 weeks of puppies’ life and every 2–3 days until approximately the month of age (Alves 2020; Kirk 2001). Overall, puppies with a birth weight under 25% of the average litter birth weight have a 12 times higher mortality rate during the first week of life (Mila et al. 2015; Mugnier et al. 2019). In addition, significant weight variations during the first 48 h of life (a loss of ≥4% of birth weight) have been reported to increase puppies’ mortality on the first 3 weeks of age (Mila et al. 2015). Weaning of puppies was performed on average at 28 days of life, and the use of milk replacer as a supplement was relatively uncommon and showed limited regional variation. This suggests that most breeders rely on maternal lactation and reserve milk replacer for indicated cases, aligning with neonatal care recommendations that caution against unnecessary supplementation.

## 5 Conclusion

Italian breeders show broadly consistent reproductive and neonatal management practices, with widespread progesterone use and a strong preference for fresh semen and natural mating. This fact might recall a low importation of frozen semen and genetic variability decreasing the genetic variability within the Italian territory. An important limitation of this study is the number of breeders involved: Although we included 251 out of approximately 1700, which would be considered a representative sample, unregistered kennels were not accounted for and could influence the overall assessment. Moreover, this study is based on self‐reported survey data and is susceptible to reporting bias. Future research should directly correlate reported practices with reproductive outcomes. These findings highlight the need for targeted educational interventions on progesterone interpretation, given that a large proportion of breeders were unaware of the P4 detection method used despite near‐universal hormonal monitoring, and on standardization of vaginal cytology reading, considering the reported variability in inter‐operator agreement. These targeted interventions may help reduce inconsistencies in reproductive management and improve breeding outcomes.

## Author Contributions

**Lluis Ferré‐Dolcet**: investigation, methodology, writing – original draft, writing – review and editing, conceptualization. **Francesco Petronella**: methodology, writing – original draft, writing – review and editing. **Giovanni Bramante**: methodology, visualization, writing – review and editing. **Alice Carbonari**: data curation, formal analysis, writing – review and editing. **Lorenza Frattina**: data curation, formal analysis, writing – review and editing. **Olga Maria Andriulo**: methodology, writing – review and editing. **Vincenzo Cicirelli**: conceptualization, methodology, writing – original draft, writing – review and editing.

## Funding

The authors have nothing to report.

## Conflicts of Interest

The authors declare no conflicts of interest.

## Data Availability

Research data are not shared.
